# Unraveling Quinoa (*Chenopodium quinoa* Willd.) Defense Against Downy Mildew (*Peronospora variabilis*): Comparative Molecular Analysis of Resistant “*Hualhuas*” and Susceptible “*Real*” Cultivars

**DOI:** 10.3390/plants13233344

**Published:** 2024-11-28

**Authors:** Walaa Khalifa, Hala Badr Khalil, Marian Thabet

**Affiliations:** 1 Department of Plant Pathology, Faculty of Agriculture, Ain Shams University, Cairo 11241, Egypt; 2 Department of Biological Sciences, College of Science, King Faisal University, Al-Ahsa 31982, Saudi Arabia; 3 Department of Genetics, Faculty of Agriculture, Ain Shams University, Cairo 11241, Egypt

**Keywords:** *Peronospora variabilis*, *Chenopodium quinoa* willd., disease resistance, disease incidence, disease severity, PR-10 protein

## Abstract

Quinoa (*Chenopodium quinoa* Willd.) is a new, promising non-conventional useful crop; however, its susceptibility to downy mildew, caused by *Peronospora variabilis*, is a key obstacle limiting its productivity in Egypt. Identifying and utilizing resistant quinoa cultivars appear to be reliable and cost-efficient strategies for controlling downy mildew, particularly in resource-limited farmers’ fields. This study aimed to evaluate the differential resistance of the Peruvian “*Hualhuas*” and Bolivian “*Real*” quinoa cultivars to *P. variabilis* infection under laboratory conditions to provide precise insight into their basic defense mechanism(s). Inoculated “*Hualhuas*” plants displayed complete resistance against *P. variabilis*, with no visible symptoms (incompatible reaction), while those of “*Real*” plants revealed high susceptibility (compatible reaction), with typical downy mildew lesions on their leaf surfaces. Disease incidence reached about 66% in the inoculated “*Real*” plants, with most inoculated leaves having lesions of grades 4 and 5 covering up to 90% of their leaf surfaces. Susceptibility indices reached up to 66% in the inoculated “*Real*” plants. Resistance to *P. variabilis* observed in the “*Hualhuas*” plants may have been largely attributed to elevated endogenous H_2_O_2_ levels, increased peroxidase (POX) activity and abundance, enhanced phenylalanine ammonia-lyase (PAL) activity and expression, as well as the upregulation of the pathogen-related protein 10 gene (*PR-10*). The results of this study indicate that the quinoa cultivar “*Hualhuas*” not only is a promising candidate for sustainable control of quinoa downy mildew but also, through a deep understanding of its molecular resistance mechanisms, would provide a possible route to enhance downy mildew resistance in other genotypes.

## 1. Introduction

Introducing resistant crop plants to agriculturally marginal environments is an important strategic solution to combat environmental hazards and reduce the population–food scarcity gap [1]. Quinoa (*Chenopodium quinoa* Willd.) is one of these crops and is increasingly gaining worldwide attention due to its high nutritive value and robust adaptability to adverse soil and agroclimatic conditions [2,3,4]. Because of its desirable agricultural traits, quinoa has been introduced and successfully established in several countries outside its native region, including the United States, Canada, Italy, Morocco, India, and Egypt, among others [5,6,7]. The integration of quinoa as a new non-conventional important crop in the cropping pattern of Egypt could increase agricultural production and improve livelihoods, particularly in very marginal and salt-prone areas [4].

Although quinoa appears to be very suitable for growth under Egyptian conditions [8], the expansion of quinoa cultivation outside its traditional growing regions might increase the spectrum of plant disease injuries [9,10]. Downy mildew, caused by *Peronospora variabilis* Gäum, is one of the most widespread and potentially destructive diseases for quinoa cultivation worldwide [11,12,13]. This pathogen belongs to the family *Peronosporaceae*, whose members are highly specialized obligate parasites (biotrophs) that parasitize vascular plants, causing downy mildew in a limited range of plant species [14]. Downy mildew of quinoa was first recorded in Peru in 1947 [15] and has since been reported in several countries around the world [11,12,13,16,17,18,19,20]. Mildew symptoms on plant foliage include dark lesions, composed of sporangia, which develop on the undersides of the leaves, causing chlorosis, necrosis, and eventually defoliation, depending on the quinoa genotype [14,21]. When the environmental conditions are conducive to downy mildew development, the infection spreads via the movement of sporangia through winds and rains, as well as by oospores that are known to remain in quinoa seeds, old leaf tissues, and the soil [22]. Downy mildew significantly reduces quinoa growth and productivity, with severe yield losses of up to 30–100%, depending on the prevailing environmental conditions, crop management practices, phenological stage of the plant, and degree of plant resistance [12,23,24,25].

Controlling downy mildew using agrochemicals is a non-sustainable approach due to its environmental hazards and may eventually be overcome by resistant isolates, as the pathogen is sexually recombinant [23,26,27]. Hence, identifying and utilizing resistant (or partially resistant) quinoa cultivars appears to be a more reliable and cost-efficient approach to managing downy mildew, particularly in resource-poor farmers’ fields. Quinoa demonstrates a high degree of resistance against *P. variabilis*, as shown by many comparative studies on various accessions, landraces, and cultivars [13,28,29,30]. Valley quinoa cultivars growing in regions with high humidity and rampant disease often display high to moderate mildew resistance, while those of southern Altiplano, growing in drier regions, show more susceptibility to downy mildew [9,31]. In a previous study, data on disease incidence, severity, and susceptibility indices showed that the lowland quinoa cultivar “*Hualhuas*” was completely resistant to downy mildew compared to the Altiplano quinoa cultivars “CICA” and “*Real*” under natural field conditions in Egypt [13]. In addition, a molecular analysis revealed the presence of specific ribosomal internally transcribed spacer amplicons with 866 bp (representing the ITS of *P. variabilis*) only in the diseased leaves of “CICA” and “*Real*” plants, but not in “*Hualhuas*” plants [13]. Yet, to date, the type and nature of resistance mechanisms acting in some quinoa cultivars against *P. variabilis* remain poorly understood. Characterizing these mechanisms would be useful in determining which of them could provide more durable resistance against downy mildew.

A couple of earlier studies have suggested that downy mildew resistance in quinoa is a complex trait regulated by multiple resistance genes [32,33]. As reported by Gandarillas et al. [32], the resistance of quinoa to downy mildew is governed by major genes (vertical resistance), minor genes (horizontal resistance), or a combination of both major and minor genes, leading consequently to partial or durable resistance. Horizontal resistance (also known as partial, minor gene, quantitative, or durable resistance) is the most common type, and the level of resistance varies from highly susceptible to resistant depending on the number of resistance genes that the quinoa cultivar exhibits [32]. In addition, Fondevilla et al. [33] identified 26 genomic regions linked to the traits that correlated downy mildew resistance in quinoa with accession PI614911. Several resistance genes and receptors in these regions were potential candidates for disease resistance. The functions of these genes were assigned to the disease-resistant RPP13-like protein and serine/threonine-protein kinase protein.

Host resistance to biotrophic pathogens (e.g., *P. variabilis*) involves numerous pre-existing physical as well as inducible and constitutive biochemical defense barriers [30,34]. One of the earliest defense responses activated in plant tissues upon a pathogen attack is the rapid and transient production of reactive oxygen species (ROS) [35,36,37], which include hydrogen peroxides (H_2_O_2_), hydroxyl radicals (OH^•^), and superoxide anions (O_2_^•–^). The accumulation of H_2_O_2_, the most stable of ROS, has been postulated to play a crucial role in plant defense [37,38]. In addition to its oxidative potential as an antimicrobial agent, H_2_O_2_ is involved in the cross-linking of hydroxyproline-rich cell wall glycoproteins; hypersensitive cell death; and the induction of pathogenesis-related proteins, such as salicylic acid and ethylene, as well as phytoalexins [35,37,39,40]. To eliminate the harmful effects of disease-triggered ROS, enzymic and non-enzymatic antioxidants are induced in plants upon pathogen infection [37,41]. Peroxidases (POXs) have also been proven to be involved in plant–pathogen interactions [42]. As oxidoreductive enzymes, they participate in the oxidation of phenols, suberization, and the lignification of the host plant’s cell walls during defense reactions against pathogens [43]. Phenolic compounds are also known to govern disease resistance in various plant species [44]. Phenylalanine ammonia-lyase (PAL) was the first enzyme found to be involved in the formation of various structural and defensive compounds such as lignin and phenols [45,46,47,48]. PAL has also been implicated in the biosynthetic pathway of salicylic acid, another defense-related compound and a key signaling component required for the activation of pathogen-related proteins, catalases, receptor-like protein kinases, and transcription factors [49,50]. Increased activity of PAL was observed in plants exposed to various biotic and abiotic stressors that act as resistance inducers [51]. Pathogenesis-related proteins have also been suggested to play a role in plant resistance against fungal diseases [52,53,54].

Against this background, this study was designed to compare the responses of a Peruvian (sea level ecotype) quinoa cultivar, “*Hualhuas*”, and a Bolivian (salar ecotype) cultivar, “*Real*”, to *P. variabilis* infection under laboratory conditions. These cultivars originate from different agroecological zones and have been shown to exhibit various degrees of resistance against downy mildew. Comparing the responses of these closely related cultivars to *P. variabilis* may elucidate key resistance mechanisms acting against downy mildew in quinoa and will also be significant when selecting genotypes for breeding programs.

## 2. Results

### 2.1. Disease Symptoms, Incidence, Severity, and Susceptibility Index

**Figure 1:**
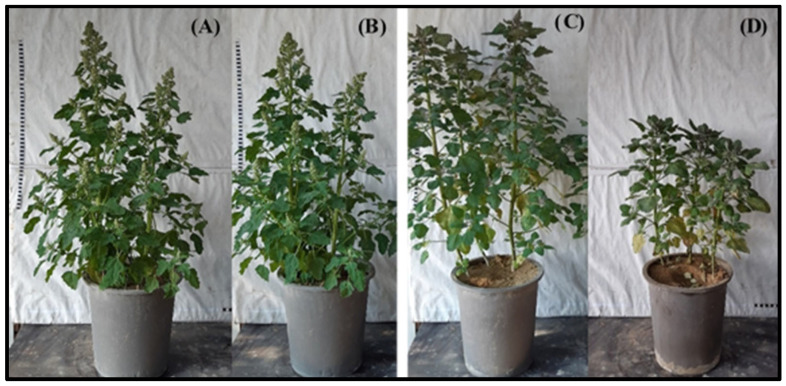
Growth and development of quinoa cultivars. Comparison of plant growth and development between the highly resistant cultivar “*Hualhuas*” [(**A**) non-inoculated controls; (**B**) inoculated plants] and the susceptible cultivar “*Real*” [(**C**) non-inoculated controls; (**D**) inoculated plants] at 21 dai with *P. variabilis*.

**Figure 2:**
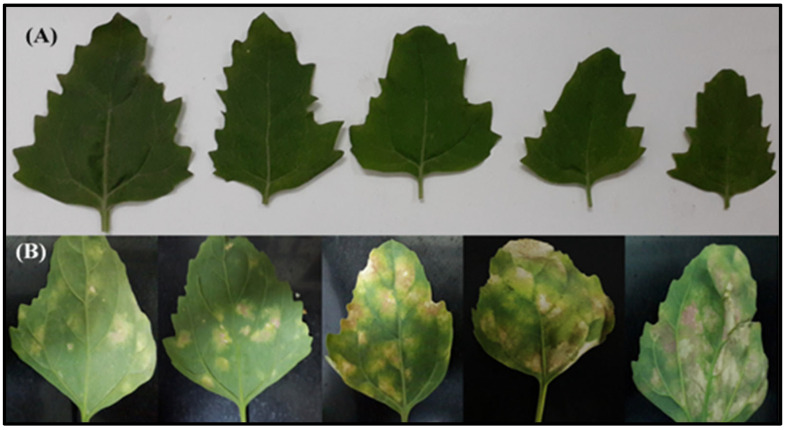
Disease symptoms on inoculated leaves of quinoa cultivars. Comparison of disease symptoms on inoculated leaves of the highly resistant cultivar “*Hualhaus*” (**A**) and susceptible cultivar “*Real*” (**B**) at 21 dai with *P. variabilis*. No symptoms of downy mildew were observed on the inoculated leaves of the highly resistant “*Hualhaus*” cultivar (**A**). In contrast, typical downy mildew symptoms, characterized by pale or yellow chlorotic lesions on the upper leaf surface and grey patches of sporangia that usually emerge on the underside of the leaves, were found on the inoculated leaves of the susceptible “*Real*” cultivar (**B**).

Inoculated plants of the *C. quinoa* var. “*Hualhuas*” showed high resistance against *P. variabilis* infection, with no visible symptoms (Figure 1B). In contrast, the inoculated plants of the *C. quinoa* var. “*Real*” showed high susceptibility, characterized by the formation of downy mildew lesions on their leaf surfaces (Figure 1D). The downy mildew symptoms on the inoculated leaves of the “*Real*” plants started as irregular chlorotic lesions on the upper surface of the adult leaves, which turned gradually into dark brown lesions, with diameters reaching up to 5 cm (Figure 2B). The corresponding lower surface of the leaves showed typical grayish-black patches of sporangia that are characteristic of downy mildew. As the infection progressed, spore formation led to a yellowish or reddish appearance in the inoculated leaves of the “*Real*” plants (Figure 2B).

**Figure 3:**
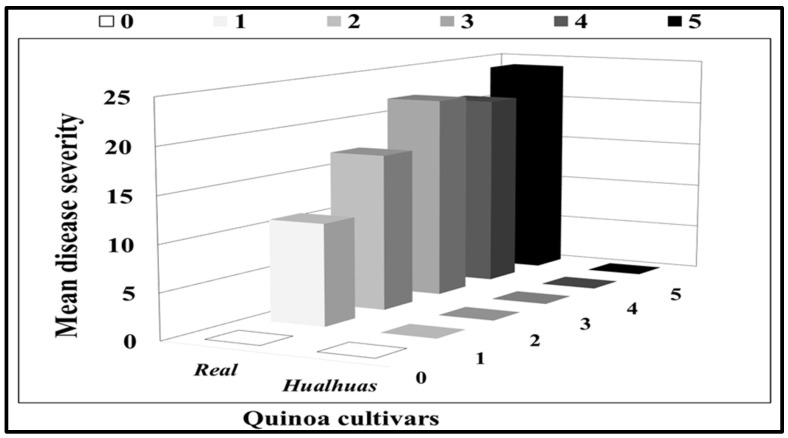
Mean disease severity of quinoa cultivars. Mean disease severity ratings for the resistant “*Hualhuas*” and susceptible “*Real*” cultivars at 21 dai with *P. variabilis*. The downy mildew symptoms on the inoculated leaves were rated from 0 to 5, as described by Mhada et al. [20]. Disease severity was calculated as the percentage of leaves in each disease category.

The disease incidence (calculated as % of sporulating leaves per plant) of the inoculated plants was 0% in “*Hualhuas*” but reached 65.86 ± 4.36% in “*Real*” (Table 1). While no downy mildew symptoms were found on the inoculated leaves of “*Hualhuas*”, visual symptomatic spots of varying sizes, colors, and sporulation degrees were observed on the inoculated leaves of the “*Real*” plants (Figure 2). Most inoculated leaves in the “*Real*” plants exhibited typical downy mildew lesions with score grades 4 and 5, which covered up to 90% of the leaf surface (Figure 3). The susceptibility indices (SI) varied among the inoculated plants, being 0% for “*Hualhuas*” and reaching 66% in the “*Real*” plants (Table 1).

### 2.2. Hydrogen Peroxide Localization and Contents

**Figure 4:**
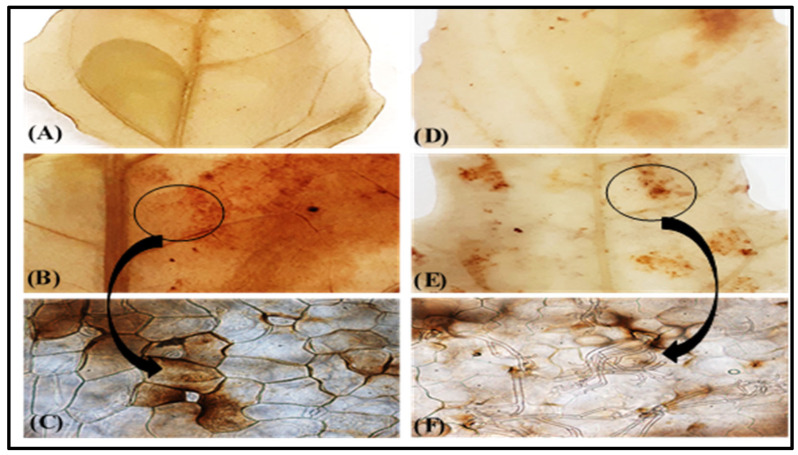
H_2_O_2_ localization in the leaf tissues. Localization of H_2_O_2_ in leaf tissues of the highly resistant cultivar “*Hualhuas*” [(**A**) non-inoculated controls; (**B**,**C**) inoculated plants] and susceptible cultivar “*Real*” [(**D**) non-inoculated controls; (**E**,**F**) inoculated plants]. Note the presence of dichotomously branched sporangiophores of *P. variabilis* with slightly curved sterigmata grown through the stomata, only in the leaves of the susceptible inoculated “*Real*” plants (**E**,**F**).

**Figure 5:**
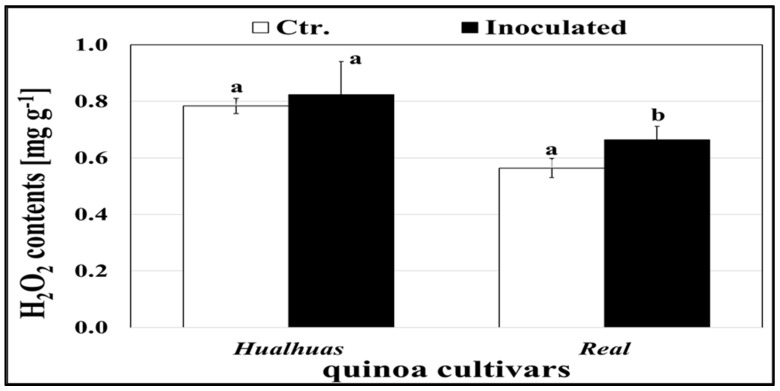
Levels of H_2_O_2_ in the quinoa leaves. Comparison of H_2_O_2_ levels in the leaves of the highly resistant “*Hualhuas*” and susceptible “*Real*” plants at 21 dai with *P. variabilis*. Each column represents the mean values from three replicates, with bars indicating standard errors. Columns sharing the same letter are not significantly different at *p* ≤ 0.05, as determined by Duncan’s multiple range test.

The hydrogen peroxide distribution in the leaves of both the healthy and inoculated quinoa cultivars is illustrated in Figure 4. Irrespective of *P. variabilis* inoculation, H_2_O_2_ accumulation was consistently higher in the leaves of the “*Hualhuas*” plants compared to those of “*Real*”, as indicated by darker reddish-brown color DAB staining (Figure 4). Light microscopic investigations revealed the presence of dichotomously branched sporangiophores extruded onto the leaf surface via the stomata, only in the inoculated susceptible cultivar “*Real*” (Figure 4F). However, the inoculated leaves of the “*Real*” plants showed comparatively greater DAB staining intensity around *P. variabilis* colonization (Figure 4F). Regardless of *P. variabilis* inoculation, the levels of endogenous H_2_O_2_ were significantly (*p* ≤ 0.05) higher in the leaves of the resistant cultivar “*Hualhuas*” compared to the susceptible cultivar “*Real*” (Figure 5). The H_2_O_2_ levels did not change significantly in the leaves of the inoculated resistant cultivar “*Hualhuas*” compared to those of the respective healthy controls (Figure 4A,B). However, higher H_2_O_2_ accumulation was triggered in the leaves of the “*Real*” plants by massive colonization of *P. variabilis* compared to their respective non-inoculated controls (Figure 4E,F). *P. variabilis* inoculation slightly enhanced H_2_O_2_ accumulation in the leaves of both cultivars by about 5.0% and 17.5% in the “*Hualhuas*” and “*Real*” plants, respectively, relative to the corresponding controls (Figure 5).

### 2.3. Enzyme Activity

**Figure 6:**
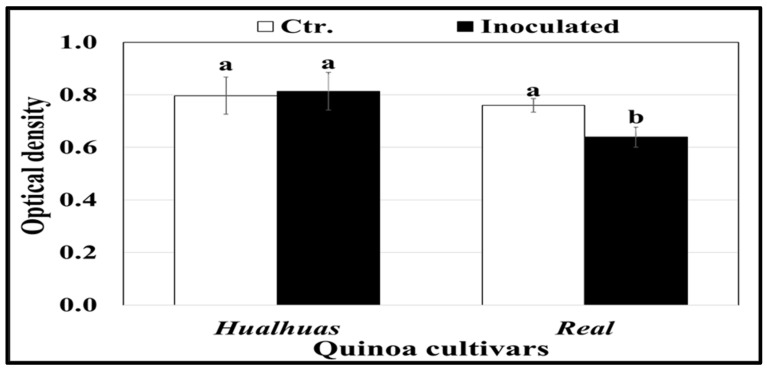
Peroxidases (POX) activity in the leaves of the highly resistant “*Hualhaus*” and the susceptible “*Real*” plants at 21 dai with *P. variabilis*. Each column represents the mean values of three replicates, and the bars represent standard errors. Columns with the same letter are not significantly different at *p* ≤ 0.05, as determined by Duncan’s multiple range test.

**Figure 7:**
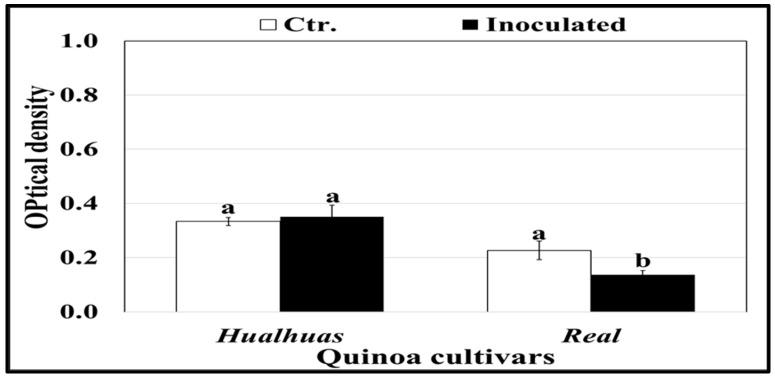
Activity of phenylalanine ammonia-lyase (PAL) in the leaves of the highly resistant “*Hualhaus*” and the susceptible “*Real*” plants at 21 dai with *P. variabilis*. Each column represents the mean values of three replicates, and the bars represent standard errors. Columns with the same letter are not significantly different at *p* ≤ 0.05, as determined by Duncan’s multiple range test.

Peroxidase (POX) activity was generally higher in the leaves of the “*Hualhuas*” plants compared to the “*Real*” ones (Figure 6). POX activity in the inoculated leaves of the “*Hualhuas*” plants was not affected but significantly (*p* ≤ 0.05) declined by roughly 16% in those of the “*Real*” plants relative to the respective controls (Figure 6). Irrespective of *P. variabilis* inoculation, phenylalanine ammonia-lyase (PAL) activity was significantly (*p* ≤ 0.05) higher in the leaves of “*Hualhuas*” than those of the “*Real*” plants (Figure 7). *P. variabilis* inoculation did not significantly alter the PAL activity of the “*Hualhuas*” plants but distinctively lowered that of the “*Real*” plants by about 39.6%, relative to the corresponding non-inoculated controls (Figure 7).

### 2.4. Expression of Quinoa Defense Genes

**Figure 8:**
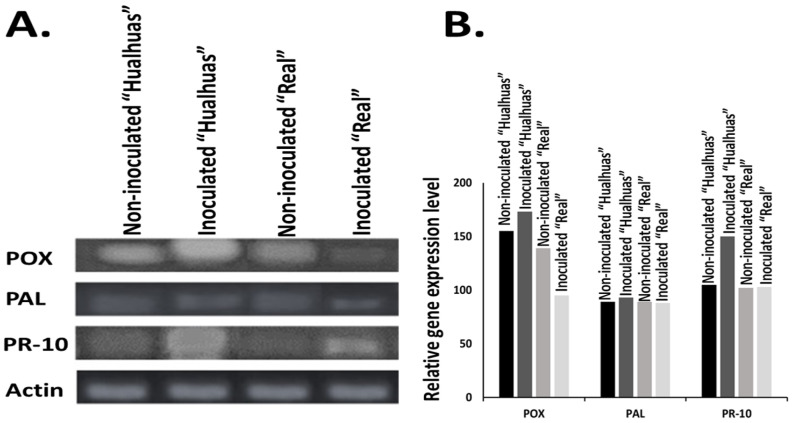
Gene expression patterns of *POX*, *PAL*, and *PR-10* in the *C. quinoa* cultivars “*Hualhuas*” (highly resistant) and “*Real*” (susceptible) following *P. variabilis* inoculation. (**A**). Gel electrophoresis showing the transcription levels of POX (200 bp), PAL (104 bp), PR-10 (217 bp), and the internal control (actin gene, 100 bp). (**B**). Bar graph illustrating the relative expression levels of the POX, PAL, and PR-10 transcripts in the non-inoculated and inoculated “*Hualhuas*” (highly resistant) and “*Real*” (susceptible) plants.

The expression levels of POX were notably upregulated in the inoculated leaves of the resistant cultivar “*Hualhuas*”, indicating a robust defense response to *P. variabilis* infection. Conversely, POX expression in the susceptible cultivar “*Real*” was remarkably downregulated, suggesting a compromised or delayed defense mechanism in response to the pathogen compared to their respective non-inoculated control plants (Figure 8A). Regarding PAL, its expression levels remained relatively stable in the inoculated leaves of the “*Hualhuas*” plants. In contrast, PAL expression in the “*Real*” cultivar was notably downregulated following inoculation with *P. variabilis*, indicating a failure to activate this critical pathway for synthesizing defense-related phenolic compounds (Figure 8A). The pathogenesis-related protein gene PR-10 exhibited strong upregulation in response to *P. variabilis* inoculation across both quinoa cultivars; however, the effect was more pronounced in “*Hualhuas*”. The elevated levels of PR-10 in “*Hualhuas*” may be part of a broader hypersensitive response, contributing to its observed resistance. Although PR-10 was also upregulated in “*Real*”, the response was comparatively weaker, potentially indicating delayed or insufficient activation of defense responses against pathogen attack (Figure 8A). This disparity in PR-10 expression between the cultivars underscores the more effective defense strategy employed by “*Hualhuas*” relative to “*Real*”.

Across all tested enzymes, the inoculated “*Hualhuas*” consistently exhibited the highest activity or expression levels, suggesting a strong inherent defense or metabolic response. Notably, *PR-10* revealed a 30% increase in transcript levels compared to the non-inoculated “*Hualhuas*” (Figure 8B). While the inoculated “*Real*” showed no differences in enzyme expression activity compared to the non-inoculated group, there was a reduction in *POX* expression (Figure 8B). This pattern suggests differential responses or resistance levels between the two cultivars, with “*Hualhuas*” demonstrating a stronger defense or metabolic response to *P. variabilis* infection.

## 3. Discussion

This study aimed to compare the response of a resistant quinoa cultivar, “*Hualhuas*”, and a susceptible cultivar, “*Real*”, to *P. variabilis* infection under laboratory conditions. Artificial inoculation provides results that can be compared to field trials, but without the varying effects of environmental factors. We intended to determine the individual physiological, biochemical, and molecular mechanism(s) conferring differences in downy mildew resistance between these closely related quinoa cultivars. This can contribute to the development of resistant quinoa cultivars in future breeding programs.

As shown in Figure 1, *P. variabilis* inoculation has stronger effects on the vitality of the susceptible cultivar “*Real*”, leading to reduced plant growth and development and altered morphology compared to the cultivar “*Hualhuas*”. Despite the favorable conditions for *P. variabilis*, the inoculated plants of “*Hualhuas*” displayed complete resistance, with no visible symptoms (incompatible reaction) (Figure 2A,B). In contrast, the *P. variabilis* inoculated plants of “*Real*” showed high susceptibility (compatible reaction), with typical downy mildew lesions on their leaf surfaces (Figure 2A,B). Downy mildew incidence reached about 66% in the inoculated “*Real*” plants, with most inoculated leaves having lesions of grades 4 and 5, covering up to 90% of their leaf surfaces (Figure 3). The calculated susceptibility indices (SI) varied in the *P. variabilis* inoculated plants, being zero for “*Hualhuas*” and reaching up to 66% in the “*Real*” plants (Table 1). These results are in qualitative agreement with our previous field screening studies and observations, confirming that the “*Hualhuas*” plants are extremely resistant, whereas the “*Real*” plants are highly susceptible to downy mildew [13].

A wide range of *P. variabilis* resistance levels has been previously demonstrated in many different accessions, landraces, and genotypes of quinoa [13,28,29,30,55]. Reportedly, the differential response of quinoa to downy mildew is strongly genotype-dependent [30,55]. It has been observed that lowland quinoa cultivars originating from the Andean valley, where humidity is high and the disease is prevalent, often display higher downy mildew resistance compared to those developed in drier regions of the high plateau [56,57]. This might explain the higher resistance to downy mildew observed in the coastal lowland cultivar “*Hualhuas*” compared to the Altiplano cultivar “*Real*” in the present study. While our study focused on a specific strain of downy mildew, the defense responses observed may reflect fundamental mechanisms that could be applicable to other strains. Future research should test additional strains to evaluate the generalizability of these findings and to explore the potential for strain-specific interactions.

Unfortunately, very little information is available regarding the precise resistance mechanisms that confer resistance in some quinoa cultivars to *P. variabilis* [26]. It is commonly assumed that downy mildew resistance in quinoa is a complex trait regulated by multiple resistance genes [30,55]. According to Gandarillas et al. [32], horizontal (broad-spectrum) resistance is the most common type of resistance against downy mildew in quinoa plants and the resistance level varies from completely resistant to highly susceptible, depending on the number of resistance genes that the quinoa cultivar exhibits. Essentially, host resistance to biotrophic pathogens involves the activation of pre-existing physical barriers as well as inducible and constitutive biochemical mechanisms [30,34]. The accumulation of H_2_O_2_ is one of the earliest host defense responses upon a pathogen attack [37,38]. It has been shown to directly inhibit fungal growth and development, thereby providing effective penetration resistance to the host plant, particularly against biotrophic pathogens (e.g., *P. variabilis*) [34,56]. In transgenic cotton, tobacco, and potato plants, elevated levels of H_2_O_2_ suppressed the disease development of several fungal genera, including *Rhizoctonia*, *Verticillium*, *Phytophthora*, and *Alternaria* [57]. Similarly, Sharma et al. [58] demonstrated that H_2_O_2_ is one of the vital defense signaling molecules that trigger resistance to *Bipolaris sorokiniana* in wheat plants. This was also evident in the present study, as *P. variabilis* inoculation induced H_2_O_2_ accumulation in the leaf tissues of both quinoa cultivars compared to the non-inoculated controls (Figure 5). Importantly, the endogenous H_2_O_2_ levels were consistently higher in the leaves of the resistant cultivar “*Hualhuas*” compared to those of the susceptible cultivar “*Real*”, as indicated by the more intense and uniform dark reddish-brown color from DAB staining in the leaf cells (Figure 4). Massive H_2_O_2_ accumulation could provide an effective penetration resistance mechanism, particularly for “*Hualhuas*” plants, further confirming their higher degree of downy mildew resistance, at least to *P. variabilis* strains currently present in Egypt. It is noteworthy that *P. variabilis* inoculation also induced H_2_O_2_ accumulation in the leaves of the “*Real*” plants, but to a lesser extent compared to the “*Hualhuas*” plants, mainly in the vicinity of the penetration attempts and around infection sites (Figure 4). Accordingly, it can be presumed that H_2_O_2_ concentrations in the tissues of the “*Real*” leaves are less effective in preventing hyphal penetration, leading consequently to an increased susceptibility in the “*Real*” plants.

Excessive levels of H_2_O_2_ and other related ROS are detrimental to many plant cell components, such as lipids, proteins, and nucleic acids [59]. To mitigate these deleterious effects, plants must regulate their antioxidant machinery through enzymatic components (such as superoxide dismutase, catalase, and peroxidase) and non-enzymatic components (such as glutathione, carotenoids, ascorbic acid, and flavonoids) [60]. Our results revealed that the activity of the POX enzyme, a well-described regulator of plant defense mechanisms, was comparatively higher in the leaves of the resistant cultivar “*Hualhuas*”, irrespective of *P. variabilis* inoculation (Figure 6). While POX activity was not changed in the inoculated leaves of the “*Hualhuas*” plants, it declined significantly by roughly 16% in those of the “*Real*” plants compared to their corresponding controls (Figure 6). Furthermore, the expression level of POX was markedly upregulated in the “*Hualhuas*” plants but downregulated in those of the “*Real*” ones upon *P. variabilis* inoculation (Figure 8A). Peroxidase induction may be associated with both H_2_O_2_ decomposition and production as well [61]. It is also involved in various plant defense reactions, such as polysaccharide bonds, the oxidation of phenols, suberization, and the lignification of cell walls during the defense reaction [62,63]. Hence, the higher expression and activity of POX observed in the resistant cultivar “*Hualhuas*” in this study might contribute to enhanced lignification, serving as a resistance mechanism against *P. variabilis*. The induction of POXs in response to pathogen inoculation was also reported in several pathosystems, and a higher increase was recorded in resistant plants compared to susceptible ones [64,65].

The accumulation of H_2_O_2_ and other related ROS is thought to trigger several orchestrated plant defense reactions, such as the induction of HR, enhanced lignin biosynthesis, and the regulation of PR gene expression [66,67,68,69,70]. During incompatible plant–pathogen interactions, pathogen recognition usually results in HR, with activation of the pathogen-induced cell death process at the sites of attack to deter biotrophic pathogens [71,72]. The occurrence of HR in quinoa has been described by several authors who noticed that resistant quinoa genotypes exhibited only a few small spots and no sporulation after *P. variabilis* infection [26,30]. However, this was not the case in the present study, as no signs typical of HR were observed in both quinoa cultivars in response to *P. variabilis* inoculation. Instead, chlorosis signs were observed only in the inoculated leaves of the susceptible cultivar “*Real*” before the pathogen was visibly sporulating from the abaxial leaf surfaces (Figure 2). The observed chlorosis in the leaves of the susceptible “*Real*” plants is likely a sign of damage, indicating a loss of cellular control over the oxidative processes; hence, a chaotic reaction occurred rather than HR. The unusual defense response observed here for both quinoa cultivars against *P. variabilis* indicates that genes implicated in the HR were not triggered and highlights the need to investigate the molecular response of quinoa to downy mildew with more details.

In many incompatible plant–pathogen systems, PAL is actively involved in the biosynthesis of many structural and defensive compounds (i.e., lignin, suberin, and other types of phenolic compounds), which are often deposited in the host cell walls at fungal invasion sites to hinder hyphal penetration [47,48]. As shown in Figure 7 and Figure 8, PAL activity and expression were slightly higher in the resistant quinoa cultivar “*Hualhuas*” compared to the susceptible one “*Real*”. Interestingly, *P. variabilis* inoculation did not significantly alter PAL activity and expression in the leaves of the “*Hualhuas*” plants but slightly reduced the activity and expression in those of the “*Real*” plants compared to their corresponding non-inoculated controls (Figure 7 and Figure 8), suggesting its involvement in the quinoa defense response against *P. variabilis*. Similarly, higher PAL activity and expression were previously documented in wheat cultivars resistant to *Fusarium graminearum* [73]. The induction of PAL activity, together with higher gene expression, has also been observed in barley plants upon powdery mildew infection [74]. The PAL activity observed in “*Hualhuas*” could be linked to the enhanced production of some compounds, contributing to its resistance to *P. variabilis*. For instance, lignin and suberin deposition strengthen the cell wall, while phenolic compounds can act directly as antifungal agents or indirectly as precursors for other defense-related pathways.

PAL has also been reported to be implicated in the biosynthesis of salicylic acid, another vital defense signaling molecule that plays a prominent role in activating PR proteins, catalases, receptor-like protein kinases, and many other transcription factors [49,50]. Several studies have shown that PR genes, including family 10 proteins, are significantly induced in response to biotic and abiotic stresses and are involved in host resistance against pathogens [75,76,77]. Our results clearly showed that the expression level of *PR-10* was markedly upregulated upon treatment with *P. variabilis* in both quinoa cultivars, the effect of which was more pronounced in the inoculated leaves of the resistant cultivar “*Hualhuas*” (Figure 8B).

These observations are consistent with previous works demonstrating that higher expression of *PR-10* in resistant plants could probably be a part of a constitutive defense mechanism. In barley, *PR-10* was markedly induced in resistant cultivars upon *Rhynchosporium secalis* infection, but not in susceptible plants [78]. Similarly, the PR-10 homologue was upregulated in the epidermal cells of resistant cowpea plants inoculated with the rust fungus *Uromyces vignae* Barclay [79].

## 4. Materials and Methods

### 4.1. Plant Materials, Growth Conditions, Experimental Setup, and Plant Inoculation

The present study was conducted in the greenhouse of the Agricultural Plant Pathology Department, Faculty of Agriculture, Ain Shams University, Qalyubia Governorate, Egypt (Latitude 30°06′42″ N; Longitude 31°14′46″ E), to unravel the physiological, biochemical, and molecular mechanisms underlying downy mildew resistance in quinoa plants under artificial conditions. The seeds of *C. quinoa* cv. “*Hualhuas*” (origin: International Potato Center, CIP, Lima, Peru) and *C. quinoa* cv. “*Real*” (origin: Salar de Uyuni, Bolivia) were surface-sterilized with 70% ethanol for 1 min and subsequently with 0.5% NaOCl for 3 min before they were rinsed thoroughly using sterile water. The seeds were then sown in black plastic pots (350 mm diameter and 250 mm height), filled with washed sand (8 kg each, dry weight basis), ten seeds per pot. The pots were kept on a bench in the greenhouse at ambient temperatures of 25 ± 3 °C during daytime and 18 ± 3.5 °C during nighttime, a photoperiod of 16 h, relative humidity of 60–70%, and light intensity of 800–1000 µmol m^−2^ s^−1^. The plants were regularly irrigated manually with a nutrient solution [80] (Hoagland and Arnon 1950) as needed. After the emergence of the first two true leaves (three weeks after germination), the plants were thinned to five seedlings of uniform size per pot. Three weeks later, the plants of each quinoa cultivar were subdivided into two groups (each of 15 plants), where they were either artificially infected with *P. variabilis* or left non-inoculated (mock controls). Each plant group was maintained in a growth chamber under temperatures of 25/20 °C during day/night, a photoperiod of 16 h, light intensity of 800–1000 µmol m^−^^2^ s^−^^1^, and a relative humidity of 80 ± 5% until the inoculation was performed.

The *P. variabilis* inoculum used in this study was prepared from the sporulating leaves of the susceptible quinoa cultivar “*Real*” grown in a naturally infected field at the Faculty of Agriculture, Ain Shams University, Qalyubia Governorate, Egypt. A solution of sporangia was prepared (on the same day the inoculation was carried out) by placing 2–3 heavily sporulating leaves in a 30 mL Falcon tube filled with 25 mL of sterilized deionized water. The tubes were gently shaken to release sporangia from the leaves. The solution was then strained through a cheesecloth and adjusted to a concentration of 4 × 105 sporangia/mL using a hemocytometer. A drop of Tween 20 was added to the inoculum to prevent the sporangia from clustering. The plants were inoculated with *P. variabilis* as described by Kitz [21] by placing humid pieces of the cheesecloth (1 cm^2^) on three successive fully expanded leaves of each plant. A total of 30 µL of the sporangia solution was pipetted onto the wet cheesecloth pieces. Downy mildew symptoms and sporulation started to occur only on the infected plants of the “*Real*” cultivar, 15–20 days after the inoculation date. The infection progressed each day as more leaves sporulated and the sporangia density increased.

### 4.2. Assessment of Disease Incidence, Severity, and Susceptibility Index

Disease incidence and severity were assessed on the leaves of both quinoa cultivars when the symptoms were fully developed on all infected plants (21 days after inoculation). Disease incidence was estimated on the leaves of ten randomly selected plants, as a percentage of infection based on the number of sporulating leaves per plant. Additionally, the leaves of the selected plants were rated for disease severity evaluation, as described by Mhada et al. [20]. The symptoms on each leaf were scored from 0 to 5, where 0 = no lesion; 1 = small lesions with a diameter less than 1 mm without sporulation on the underside of the leaves; 2 = clearly individual lesions, with a higher number and larger size (0.5–1 cm), without sporulation; 3 = lesions covering less than 50% of the leaf surface, with the beginning of sporulation at the lower side; 4 = lesions of larger size, covering more than 50% of the leaf area; and 5 = lesions covering more than 90% of the leaf area, with high sporulation on both the lower and upper leaf surfaces. Disease severity was calculated and expressed as the percentage of leaves in each category. Based on the disease severity measurements, the susceptibility index (SI) for each quinoa cultivar was calculated according to Wan et al. [81] using the following equation:

$$SI=\frac{\Sigma (grade\;value\times no.of\;leaves\;in\;that\;grade)}{total\;leaf\;number\times the\;highest\;grade\;value}\times 100$$

The downy mildew resistance level of each cultivar was scored based on its SI value, where SI = 0–5: extremely resistant (ER); 5–25: highly resistant (HR); 25–50: resistant (R); 50–75: susceptible (S); and >75: highly susceptible (HS) according to Staudt and Kassemeyer [82].

### 4.3. Histochemical Localization of Hydrogen Peroxide (H_2_O_2_)

The detection of hydrogen peroxide (H_2_O_2_) localization was performed using 3,3-diaminobenzidine (DAB, Sigma-Aldrich, St. Louis, MO, USA), as described by Shetty et al. [83]. Representative leaves from the plants of each treatment of both quinoa cultivars were collected at 21 dai, and the segments were incubated in DAB solution for 12 h. They were then fixed and decolorized in a boiling 95% ethanol solution for 10 min, before being cleared in a saturated chloral hydrate solution. Leaf segments were then mounted on glass slides using a 50% glycerol solution and examined microscopically using a Leica DM 2500 light microscope. Reddish-brown staining in the leaf tissues at the penetration sites indicated H_2_O_2_ accumulation.

### 4.4. Hydrogen Peroxide (H_2_O_2_) Content

Hydrogen peroxide (H_2_O_2_) content in the leaves of both quinoa cultivars was determined according to the methods of Junglee et al. [84]. At the harvest time, approximately 0.1 g of fresh leaf material was ground into a fine powder in liquid nitrogen, and a 1.5 mL solution containing 10 mM phosphate buffer (pH 6.5), 0.1% Trichloroacetic acid (TCA), and 1 M potassium iodide (KI) was added directly to the frozen leaf tissue powder. A control was prepared with H_2_O instead of KI. All samples were kept protected from light at 4 °C for 10 min. They were then centrifuged at 12,000 rpm for 15 min at 4 °C. The absorbance of the supernatant was then measured at 390 nm using a spectrophotometer (Unico-2100, Monmouth County, NJ, USA). A calibration curve obtained with H_2_O_2_ standard solutions prepared in 0.1% TCA was used.

### 4.5. Extraction and Assay of Peroxidase (POX) Activity

The extraction was performed according to Biles and Martyn [85] as follows: 1 g of leaf tissues (21 dai) was ground into a fine powder in liquid nitrogen, and a 2 mL sodium phosphate buffer (0.1 M, pH 6.5) was added to the frozen leaf materials. The extracts were transferred to 2 mL Eppendorf tubes and centrifuged for 20 min at 12,000 rpm at 4 °C. Peroxidase activity was directly determined in the supernatant of the crude enzyme extracts, according to Hammerschmidt et al. [86]. The reaction mixture consisted of 2.9 mL of a 100 mM sodium phosphate buffer (pH 6.0) containing 0.25% (*v*/*v*) guaiacol (2-Methoxyphenol) and 100 mM H_2_O_2_. The reaction was started by adding 100 µL of the crude enzyme extract. The changes in absorbance were recorded for 3 min at 470 nm. Enzyme activity was expressed as the increase in absorbance min^−^^1^ g^−^^1^ fresh weight using a spectrophotometer (Unico-2100).

### 4.6. Extraction and Assay of Phenylalanine Ammonia-Lyase (PAL) Activity

Phenylalanine ammonia-lyase (PAL) activity was determined according to Solecka and Kacperska [87]. Approximately 1 g of leaf tissues (21 dai) was ground into a fine powder using liquid nitrogen, and a 2 mL sodium borate buffer (50 mM, pH 8.8) was added to the frozen leaf materials. The extract was transferred to 2 mL Eppendorf tubes and centrifuged for 20 min at 12,000 rpm at 4 °C. The supernatant was used as a source of crude enzyme for the assay of the PAL activity. The reaction mixture consisted of 1 mL of crude enzyme solution, 2 mL of sodium borate buffer (50 mM, pH 8.8), and 1 mL of 10 M of L-phenylalanine. Incubation was performed at 30 °C for 1 h, and the reaction was terminated by adding 500 µL of HCl (6N). The reaction mixture was then centrifuged for 10 min at 12,000 rpm. PAL activity was expressed as trans-cinnamic acid released, measured at 290 nm using a spectrophotometer (Unico-2100).

### 4.7. Gene Expression Levels of PAL, POX, and PR-10

To further verify the resistance mechanism of quinoa against downy mildew, the transcriptional levels of PAL, POX, and one of the pathogenesis-related proteins (PR-10) were investigated using RT-PCR. The actin gene was used as a reference gene to standardize the expression levels of all genes [13]. Representative leaf samples were collected, and the total RNA was extracted using the Jena Bioscience Purification Kit according to the manufacturer’s instructions (Jena, Germany). To obtain cDNA, mRNA was amplified using a one-step RT-PCR kit (QIAGEN, Hilden, Germany) following the manufacturer’s protocol. The gene-specific primers for cDNA amplification are listed in Table 2. Reverse transcription was performed in a thermal Eppendorf master cycler (T100TM thermal cycler, BIO-RAD, Hercules, CA, USA) at 50 °C for 30 min, followed by PCR amplification for 35 cycles of denaturation at 94 °C for 1 min (15 min for the first cycle), annealing at 55 °C for 1 min, and extension at 72 °C for 1 min, with a final extension at 72 °C for 10 min. After completion of the reaction, the amplification products were visualized on a 1% agarose gel using Tris-acetate-EDTA (TAE) buffer and stained with ethidium bromide (EB).

### 4.8. Gel Electrophoresis Image Analysis Method

The gel electrophoresis image was analyzed using GelAnalyzer software (GelAnalyzer 23.1, http://gelanalyzer.com, accessed on 15 November 2024). The analysis process involved several steps, including automatic lane detection, band identification, and quantification. The software allows for background correction and distortion adjustment to improve accuracy.

### 4.9. Statistical Analysis

All data sets were subjected to analyses of variance (ANOVAs) using Duncan’s multiple range test from the SPSS 16.0 statistical package (SPSS, Chicago, IL, USA) to find a posteriori homogeneous sub-groups of means that differ significantly at *p* ≤ 0.05.

## 5. Conclusions

In conclusion, the results obtained throughout this study complement and confirm our previous findings, clearly demonstrating that the lowland cultivar “*Hualhuas*” exhibits extreme resistance to downy mildew compared to the Altiplano cultivar “*Real*”. This resistance can be largely attributed to the higher endogenous H_2_O_2_ levels, increased peroxidase activity and abundance, elevated PAL activity and expression, and enhanced PR-10 gene expression in the inoculated leaves of the “*Hualhuas*” plants compared to those of “*Real*”. It is important to note that the degree of resistance against *P. variabilis* reported here for the cultivars “*Hualhuas*” and “*Real*” may vary with a different isolate of *P. variabilis*. Finally, the results of this study allow for the speculation that the Peruvian quinoa cultivar “*Hualhuas*” not only is a promising candidate for the sustainable control of quinoa downy mildew under our conditions but also, through a deep understanding of its molecular resistance mechanisms, may provide a potential route to enhance downy mildew resistance in other genotypes.

## Figures and Tables

**Table 1:** Disease incidence and susceptibility index of the resistant “*Hualhuas*” and susceptible “*Real*” cultivars at 21 dai with *P. variabilis*.

**Cultivar**	**Treatments**	**Disease Incidence**	**Susceptibility Index**
*Hualhuas*	Non-inoculated	0.000 ± 0.000	0.000 ± 0.000 ^a^
	Inoculated	0.000 ± 0.000 ^a^	0.000 ± 0.000 ^a^
*Real*	Non-inoculated	0.000 ± 0.000 ^a^	0.000 ± 0.000 ^a^
	Inoculated	65.863 ± 4.363 ^b^	66.015 ± 0.264 ^b^

Each value represents the mean of 10 replicates. Means within a column followed by the same letter are not significantly different at *p* ≤ 0.05, as determined by Duncan’s multiple range test.

**Table 2:** Primers used for RT-PCR in this study.

Gene	Forward Primer (5’ to 3’)	Reverse Primer (5’ to 3’)
*POX*	GGTCAGGTAATCCAGTGTTGC	GCTCTCCGGGGCTCAC
*PAL*	AAGCTGATGTTCGCGCAGTTCT	AAACCATAGTCCAAGCTCGG
*PR10*	AAGGAGATGTTCTTGGAGACAAACTTG	AGCGTAGACAGAAGGATTGGCG
*Actin*	TCATACGGTCAGCAATAC	ATGTGGATATCAGGAAGGA

## Data Availability

The data sets generated during the current study are available from the first author upon reasonable request.
